# Initial characterization of an iron superoxide dismutase from *Thermobifida fusca*

**DOI:** 10.1007/s00775-023-02019-9

**Published:** 2023-09-19

**Authors:** Anne Grethe Hamre, Rim Al-Sadawi, Kirsti Merete Johannesen, Bastien Bisarro, Åsmund Røhr Kjendseth, Hanna-Kirsti S. Leiros, Morten Sørlie

**Affiliations:** 1 Department of Chemistry, Biotechnology and Food Science, Norwegian University of Life Sciences, PO 5003, 1432 Ås, Norway; 2 Department of Chemistry, Faculty of Science and Technology, UiT The Arctic University of Norway, 9037 Tromsö, Norway; 3 Department for Physics and Technology, Faculty of Science and Technology, UiT The Arctic University of Norway, 9037 Tromsö, Norway

**Keywords:** Superoxide dismutase, Thermobifida fusca, Enzyme thermostability, Crystal structure

## Abstract

Superoxide dismutases (SODs) are enzymes that catalyze the dismutation of the superoxide radical anion into O_2_ and H_2_O_2_ in a two-step reaction. They are ubiquitous to all forms of life and four different types of metal centers are detected, dividing this class of enzymes into Cu-/Zn-, Ni-, Mn-, and Fe-SODs. In this study, a superoxide dismutase from the thermophilic bacteria *Thermobifida fusca* (*Tf*SOD) was cloned and expressed before the recombinant enzyme was characterized. The enzyme was found to be active for superoxide dismutation measured by inhibition of cytochrome *c* oxidation and the inhibition of the autoxidation of pyrogallol. Its pH-optimum was determined to be 7.5, while it has a broad temperature optimum ranging from 20 to 90 °C. Combined with the *T*_m_ that was found to be 78.5 ± 0.5 °C at pH 8.0, *Tf*SOD can be defined as a thermostable enzyme. Moreover, the crystal structure of *Tf*SOD was determined and refined to 1.25 Å resolution. With electron paramagnetic resonance spectroscopy, it was confirmed that iron is the metal co-factor of *Tf*SOD. The cell potential (*E*_m_) for the TfSOD-Fe^3+^/TfSOD-Fe^2+^ redox couple was determined to be 287 mV.

**Graphical abstract:**

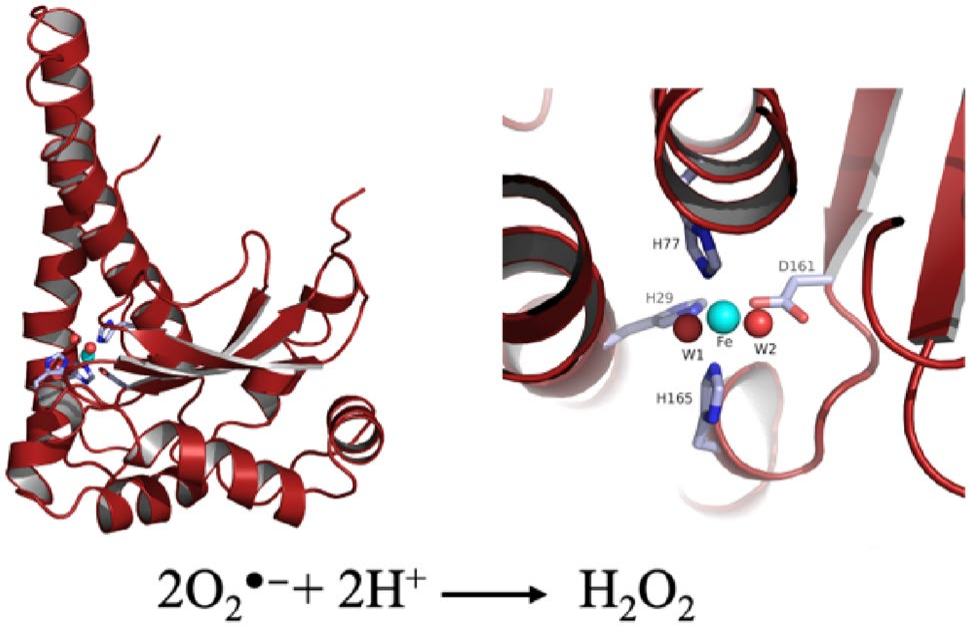

**Supplementary Information:**

The online version contains supplementary material available at 10.1007/s00775-023-02019-9.

## Introduction

Reactive oxygen species (ROS), such as the superoxide radical (O_2_^·^ ®), hydrogen peroxide (H_2_O_2_), and the hydroxyl radical (OH^·^), are normal by-products of cellular metabolism and can lead to oxidative stress to the cells by reacting with and damaging intracellular targets such as lipids, proteins and DNA. Superoxide dismutases (SODs, EC 1.15.1.1) are metal enzymes that play a major role in detoxifying these harmful oxygen species. They catalyze the disproportionation of superoxide radicals into oxygen (O_2_) and hydrogen peroxide (H_2_O_2_) [1] in a two-step reaction in which the metal ion cycles between two oxidation states;

1 $${\mathrm{O}}_{2}^{\bullet -}+{\mathrm{M}}^{3+}\to {\mathrm{O}}_{2}+{\mathrm{M}}^{2+}$$

2 $${\mathrm{O}}_{2}^{\bullet -}+{\mathrm{M}}^{2+}+2{\mathrm{H}}^{+}\to {{\mathrm{H}}_{2}\mathrm{O}}_{2}+{\mathrm{M}}^{3+}$$

ROS-induced oxidative stress has been implicated in the pathophysiology of several conditions, e.g., aging, cardiovascular diseases, neurological disorders, transplant rejection, asthma, rheumatoid arthritis, and cancer. SOD has been shown to be able to reverse or prevent unfortunate effects of several of these conditions [2]. In addition, SODs are widely used in cosmetics, health care products, agriculture as well as pharmaceuticals due to their generally vast bioavailability, high affinity, and high elimination rates of ROS [3, 4]. They also confer protection against ROS-induced oxidative stress in microorganisms [2]. SODs are also linked to depolymerization of lignocellulose, both as a catalyst for direct oxidation of lignin [5], as well as supplier of H_2_O_2_ as a co-substrate for cellulose oxidation by lytic polysaccharide monooxygenases (LPMOs) [6]. For LPMO catalyzed oxidation of cellulose, this is important since it has been shown that production of H_2_O_2_ is the limiting step in the catalysis [7–9], and that high catalytic efficiency of LPMOs at low concentrations of H_2_O_2_ can take place without enzyme deactivation [10]. The controlled use of SOD is shown to provide sufficient amount for cellulose oxidation by LPMOs without enzyme inactivation [6].

The name SOD comprises four distinct enzyme groups being related to the protein fold and the metal cofactor they employ to mediate their chemistry. The groups are copper/zinc SOD (Cu/Zn-SOD), nickel SOD (Ni-SOD), and the highly homologous manganese and iron SOD (Mn-SOD and Fe-SOD). All four groups are found in both eukaryotic and prokaryotic organisms [11]. In prokaryotes, Cu/Zn-SODs are commonly found in the periplasm [12, 13], Fe-SODs in the cytoplasm, and Mn-SODs in both the periplasm and cytoplasm [14–17].

Despite varying catalytic metals, common to all groups is their general disproportionation reaction. However, the different groups have distinct protein architectures. Ni-SODs are described as homohexamers of four-helix bundles with a total weight of approximately 80 kDa [18–20]. In Cu/Zn-SODs, the main structural motif is a flattened eight-strand β-barrel. Typically, these enzymes are found as homodimers of approximately 32 kDa [20, 21]. Single Cu-SODs also exist [22–24]. A common feature of these are an unusual open-access active site and the lack of an electrostatic loop that guides the substrate to the active site. The Mn- and Fe-SODs are structurally similar, normally appearing as 45 kDa homodimers where each monomer has an α-helical N-terminal domain and a C-terminal domain comprised of a three-stranded β-sheet surrounded by α-helices [20, 25, 26].

The active site of Mn- and Fe-SODs is located in an area occupied by a short and variable helix. The metal ion in each monomer is coordinated in a highly conserved, strained trigonal bipyramidal geometry by three histidine residues, one aspartate residue, and one water molecule or HO^−^ ion [27]. The superoxide reaches the active site through a funnel with a narrow opening, using electrostatics for guidance [27, 28]. A few cambialistic SOD can fulfill their function with both Fe^2+/3+^ and Mn^2+/3+^ as cofactors [29].

Different SODs from a diverse group of organisms, including psychrophilic, mesophilic and thermophilic bacteria have been characterized [30–33]. The thermophilic soil bacterium *Thermobifida fusca* underwent an extensive label free quantitative proteomic analysis of its secretome after grown on different lignocellulosic biomasses [34]. Among the hemicellulose, pectin and lignin degrading enzymes showing induced expression, a superoxide dismutase, likely an Fe-SOD, was detected [34]. Moreover, in a study conducted by Rashid et al*.* in 2015, two MnSODs from the thermotolerant *Sphingobacterium *sp*.* T2 were found to show high activity for oxidation of Organosolv and Kraft lignin, as well as lignin model compounds, generating multiple oxidation products [5].

*T. fusca* is an actinomycete that appears to be a major degrader of plant cell walls in heated organic material such as compost piles and rotting hay [35]. It holds a high biocatalytic potential as it serves as a source for several highly thermostable enzymes; e.g., catalase, Baeyer–Villiger monooxygenase, and several different glycoside hydrolases [36–39]. For industrial applications, it is preferable that an enzyme has both structural and functional stability under severe conditions. The thermostability is one of the most important properties, offering robust catalyst alternatives, being able to withstand the often relatively harsh conditions of industrial processing, e.g., in biorefineries [40]. In biorefineries, renewable resources such as agricultural crops or wood are utilized for extraction of intermediates or for direct conversion to chemicals, commodities or fuels [41, 42]. High temperatures can promote better enzyme penetration and cell-wall disorganization of the raw materials [43].

Here, we have cloned, expressed, solved the crystal structure, and performed an initial characterization of the superoxide dismutase (*Tf*SOD) expressed in the proteomic analysis conducted by Adav et al. (Accession number: gi|72161361, Uniprot entry: Q47RC2). Our results show that *Tf*SOD do have iron as a cofactor and indeed is a thermostable enzyme making this a candidate for the use in modern biorefinery setups.

## Materials and methods

### Chemicals

Protease inhibitor cocktail tablets were purchased from Roche (Basel, Switzerland). All other chemicals were of analytical grade and purchased from standard manufacturers.

### Enzyme expression and purification

#### Cloning

The gene encoding *Tf*SOD (Uniprot ID: Q47RC2) was codon optimized and cloned into the pET-22b(+) vector by Genscript (Piscataway, NJ, USA). Received plasmids were transformed into *Escherichia coli* BL21Star (DE3) cells (Life Technologies, Carlsbad, CA, USA).

#### Protein expression

For protein expression, *E. coli* BL21(DE3) cells containing the *Tf*SOD plasmid were inoculated into 50 ml LB medium containing 100 µg/ml ampicillin and grown at 37 °C and 200 rpm for 20 h. The cell culture was then inoculated into 1 L TB medium containing 100 µg/ml ampicillin and cultivated at 30 °C until the OD_600_ reached 0.6–0.8. The temperature was decreased to 18 °C, and gene expression was induced with 0.2 mM isopropyl-β-d-thiogalactopyranoside (IPTG) for 20 h. The cells were then harvested by centrifugation (8000 rpm, 20 min at 4 °C).

Cell pellets were resuspended in lysis buffer (50 mM Tris–HCl pH 8.0, 1 mM EDTA, protease inhibitor cocktail tablets, and 0.3 mg/ml lysozyme) before 1 h incubation at 30 °C. The cells were then lysed by sonication (4 min, 5 s interval), and the cell debris was removed by centrifugation (8000 rpm, 30 min at 4 °C). The supernatant was collected, and the volume measured (45 ml). An 5 ml/10 10% w/v streptomycin sulfate solution (adjusted to pH 7 with 2.5% NH_3_) was added dropwise to the supernatant with careful stirring over a period of 5 min before the solution was incubated for 10 min at room temperature. The solution was then centrifuged for 20 min at 4 °C and 8000 rpm. The supernatant was thereafter sterilized by filtration (0.2 µm) and stored at 4 °C prior to purification.

#### Protein purification

*Tf*SOD was purified using a two-step protocol including ion exchange chromatography (IEC) and hydrophobic interaction chromatography (HIC). The supernatant (“Protein expression”) was adjusted to pH 8.0 and loaded onto a HiTrap Q HP column (5 ml) (GE Healthcare) connected to a BioLogic low-pressure protein purification system (Bio-Rad, Hercules, CA, USA). *Tf*SOD was eluted by applying a linear salt gradient (0–600 mM NaCl) over 20 column volumes (100 ml) at a flow rate of 4 ml/min. The *Tf*SOD containing fractions were pooled and adjusted to buffer A (50 mM Tris–HCl pH 8.0, 1 M (NH_4_)_2_SO_4_) with 3 M (NH_4_)_2_SO_4_ and loaded onto a HiTrap PhenyL HP column (5 ml) (GE Healthcare) connected to a BioLogic low-pressure protein purification system (Bio-Rad). *Tf*SOD with a purity > 95% eluted in the flow through with a flow rate of 4 ml/min.

Protein purity was analyzed by sodium dodecyl sulfate–polyacrylamide gel electrophoresis after each purification step. After the last step, pure protein was concentrated and the buffer changed to either 50 mM sodium phosphate pH 7.0- or 50-mM Tris–HCl pH 7.5 using Macrosep^®^ Advances Centrifugal Devices with a 10 kDa cutoff (Pall laboratories, Port Washington, NY, USA). The protein concentration was determined by absorbance at A_280_, using the theoretical extinction coefficient 51,910 and the molecular weight 22,716 Da [44].

### Determination of enzyme activity

#### Inhibition of pyrogallol autoxidation

Inhibition of pyrogallol autoxidation was measured in 50 mM Tris–HCl pH 8.2 containing 1 mM EDTA at 325 nm [45, 46]. The reactions were run in a total volume of 300 µl in an Ultra-micro rectangular 10 mm cell, using an Agilent Cary 8454 UV–Visible spectrophotometer (Agilent Technologies, Santa Clara, CA, USA). First, the autoxidation process was monitored at 325 nm by mixing the buffer/EDTA solution with 0.2 mM pyrogallol. The absorbance was measured every 30 s over a period of 4 min. Second, *Tf*SOD was added to the reaction, and the inhibition of the autoxidation was monitored under the same conditions and time-period as for the autoxidation. All samples were run in triplicates. The amount of *Tf*SOD giving an inhibition of 50% was taken as 1 Unit of enzyme, and Eq. 3 shows how the activity was calculated.

3 $$\mathrm{Activity }\left(\frac{U}{\mathrm{mL}}\right)=\frac{\frac{\Delta {A}_{325\mathrm{ blank}}-\Delta {A}_{325\mathrm{ sample}}}{\Delta {A}_{325\mathrm{ blank}}}\bullet 100\mathrm{ \%}}{50\mathrm{ \%}}\bullet 0.3\bullet \frac{1}{V}\bullet D$$

where $\Delta {A}_{325 \mathrm{blank}}$ is the autoxidation rate determined in the blank, $\Delta {A}_{325 \mathrm{sample}}$ is the autoxidation rate in the sample, 0.3 is the total volume of the reaction in ml, *V* is the volume of sample in ml, and *D* is the dilution factor of the sample.

#### Inhibition of cytochrome *c* reduction

Inhibition of cytochrome *c* reduction was measured in a cocktail of 50 mM sodium phosphate pH 7.8 containing 0.1 mM EDTA, 50 µM xanthine, and 10 µM cytochrome *c* [1]. The reactions were run in a total volume of 3.00 ml in a rectangular 10 mm cell, using an Agilent Cary 8454 UV–Visible spectrophotometer (Agilent Technologies). The absorbance of all samples was monitored at 550 nm every 10 s over a period of 2 min. First, a blank sample containing the above-mentioned cocktail was monitored. Second, xanthine oxidase was added to the cocktail in an amount giving a change in absorbance of 0.025 ± 0.005 per minute, this being an uninhibited sample. Finally, an inhibited sample was monitored by adding *Tf*SOD to the cocktail containing xanthine oxidase.

### pH optimum

The pH optimum was determined by the pyrogallol autoxidation method described in “Inhibition of cytochrome c reduction” using 50 mM Tris–HCl (pH 7.0, 7.5, 8.0, 8.5, and 9.0), 1 mM EDTA, 0.2 mM pyrogallol, and 0.4 µM *Tf*SOD. The absorbance was measured every 20 s over a period of 4 min. The degree of inhibition was calculated for each pH-value, and the degree of inhibition at optimal pH was taken as 100%.

### Temperature optimum

The temperature optimum was determined by the pyrogallol autoxidation method described in “Inhibition of cytochrome c reduction”. The degree of inhibition was assessed at 25, 30, 40, 50, 60, 70, 80 and 90 °C. The degree of inhibition was calculated for each temperature, and the degree of inhibition at optimal temperature was taken as 100%.

### Fluorescence–based protein thermal stability assay

The thermal stability of *Tf*SOD was determined using a fluorescence-based thermal stability assay in an MJ minicycler (Bio-Rad) [47]. The assay volume used was 25 µl, which included (final concentrations) 0.50 mg/ml enzyme, 300X SYPRO Orange solution from a 5000X stock solution, and 100 mM buffers at pH 5.0 (Sodium acetate), pH 6.0 (MES), pH 7.0 (Tris–HCl), pH 8.0 (Tris–HCl), pH 9.0 (Bicine), and pH 10.5 (CHAPS). The temperature gradient was from 10 to 95 °C with an increase of 1 °C per minute. The melting temperature (*T*_m_) was determined to be the inflection point of the melting transition found from the first derivative. All experiments were performed in duplicate.

The principles behind the fluorescence-based thermal stability assay are that the fluorescence dye (SYPRO Orange) binds the hydrophobic residues that gets exposed during unfolding and give the fluorescence signal. At temperatures higher than the fluorescence peak, the protein aggregates, and the fluorescence signal drop due to lack of dye to protein interactions.

### Determination of the midpoint potential (*E*_m_) for the TfSOD-Fe^3+^/TfSOD-Fe^2+^-redox couple

Solutions (50 μl) of oxygen-free *N*,*N*,*N*′,*N*′-tetramethyl-1,4 phenylenediamine (TMP_red_) in its reduced form (200 μM) and *Tf*SOD-Fe^*3*+^ (70 μM) in 50 mM Tris–HCl (pH 8.0, *t* = 25 °C) were mixed in a cuvette and placed in a Hitachi U-1900 spectrophotometer. The extent of reaction was determined by measuring absorbance from the formed TMP radical cation (TMP_ox_) at *λ* = 610 nm, and concentrations of TMP_ox_, which equal concentrations of *Tf*SOD-Fe^2+^ (Eq. 4), were calculated by using an extinction coefficient of 14.0 mM^−1^ cm^−1^ [48].

4 $${\mathrm{TMP}}_{\mathrm{red}}+{Tf{\mathrm{SODFe}}}^{3+} \rightleftarrows {\mathrm{TMP}}_{\mathrm{ox}}+{Tf{\mathrm{SODFe}}}^{2+}$$

From the determined concentrations (TMP_ox_ and *Tf*SOD-Fe^2+^), the equilibrium constant (*K*) was calculated (Eq. 5).

5 $$K=\frac{\left[{\mathrm{TMP}}_{\mathrm{ox}}\right]\left[{\mathrm{TfSOD}}^{{\mathrm{Fe}}^{2+}}\right]}{\left[{\mathrm{TMP}}_{\mathrm{red}}\right]\left[{\mathrm{TfSOD}}^{{\mathrm{Fe}}^{3+}}\right]}$$

The relationship between the free energy change (*ΔG*_*r*_*°*), the equilibrium constant (*K*), and the cell potential (*E*^*°*^) is shown in Eq. 6.

6 $$\Delta {G}_{r}^{\mathrm{o}}=-RT\mathrm{ln}K=-nF{E}^{\mathrm{o}}$$

where *R* is the gas constant, *T* is the temperature in Kelvin, *n* is the number of electrons transferred in the reaction, and *F* is the Faraday constant. The midpoint potential for the *Tf*SOD-Fe^3+^/*Tf*SOD-Fe^2+^ redox couple was determined by adding the known cell potential of 273 mV for TMP_red_/TMP_ox_ to the cell potential of the equilibrium reaction of TMP_red_ and *Tf*SOD-Fe^3+^ [49, 50].

### Crystallization and structure determination

Crystallization trials for *Tf*SOD (2.8 mg/ml) in 50 mM Tris–HCl pH 7.5 were set up with the sitting-drop method using a Phoenix DT crystallization robot (Rigaku) using drops of 500 nl protein and 500 nl reservoir solution in 96-well MRC plates (Molecular Dimensions) with 60 µl reservoir volume. About 570 drops were put up using commercial and in-house made screens, and several crystals appeared. The best quality crystal was grown from 25% (w/v) PEG 1500 and 0.1 M sodium malonate dibasic monohydrate, imidazole and boric acid buffer at pH 8.0, which is from a PACT premier screen [51]. The crystal was flash frozen directly in liquid nitrogen without any cryo-additives.

The X-ray data collection was performed at beamline BL14.1 at BESSY, Berlin, Germany, and integrated, scaled and truncated in iMOSFLM [52] and AIMLESS [53].

A homology model was made using SOD from *Propionibacterium freudenreichii* (PDB ID: 1BSM; 73% sequence identity) and the *Tf*SOD sequence in the program SWISS-MODEL [54]. A monomer of this model was used to solve the *Tf*SOD structure by molecular replacement in the PHASER crystallographic software [55]. The structure was refined in the Phenix software [56] with manual rebuilding in the graphical program WinCoot [57]. All structural figures were made using PyMol [58].

### Electron paramagnetic resonance (EPR)

A sample of 200 μM *Tf*SOD in 50 mM sodium phosphate pH 7.0 was prepared in a Wilmad quartz EPR tube. The EPR spectrum was recorded using a BRUKER EleXsyS 560 SuperX instrument equipped with an ER 4122 SHQE SuperX high-sensitivity cavity and a liquid nitrogen cooled cold finger. The spectrum was recorded at 77 K at a microwave frequency of 9.4196 GHz using a microwave power of 1.0 mW and a modulation amplitude of 10 G. EasySpin (REF: https://doi.org/10.1016/j.jmr.2005.08.013) was used to simulate and estimate the g-values in the observed EPR spectrum.

## Results and discussion

In this work, the gene encoding *Tf*SOD was, therefore, codon optimized and cloned into the pET-22b(+) vector. Plasmids were transformed into *E. coli* BL21Star (DE3) cells and overexpressed in TB-amp medium at 18 °C for 20 h after induction with 0.2 mM IPTG. Purification of *Tf*SOD was achieved through a two-step protocol using strong anion exchange chromatography and hydrophobic interaction chromatography. In the HIC-step, pure *Tf*SOD eluted in the flow through giving a yield of 7 mg/l culture.

Many different SODs, from various groups of organisms have been characterized. Among those are several produced by thermophilic bacteria and fungi, e.g. *Thermus thermophiles, Thermus filiformis, Thermosynechococcus elongatus, Thermotrix sp, Thermomyces lanuginosus,* and *Chloroflexus aurantiacus* [60–64]. To determine the *Tf*SOD activity as well as its pH- and temperature optimum, the method based on the inhibition of pyrogallol autoxidation by SOD was used [45, 46] (Fig. S1). However, this method is limited in its pH range since pyrogallol only autoxidizes in alkaline solutions [65]. Using this method, *Tf*SOD was found to be active for superoxide dismutation. At standard assay conditions (room temperature and pH 8.2), the activity was determined to be 124 U/mg when a concentration of 0.67 μM of *Tf*SOD was used. This is in the same range as the two lignin active MnSODs from *Sphingobacterium sp.* T2 that showed specific activities of 400 U/mg and 124 U/mg, respectively [5]. As a qualitative control, *Tf*SOD was also found to be active for superoxide dismutation measured by inhibition of cytochrome *c* oxidation (results not shown) [1, 66].

**Fig. 1:**
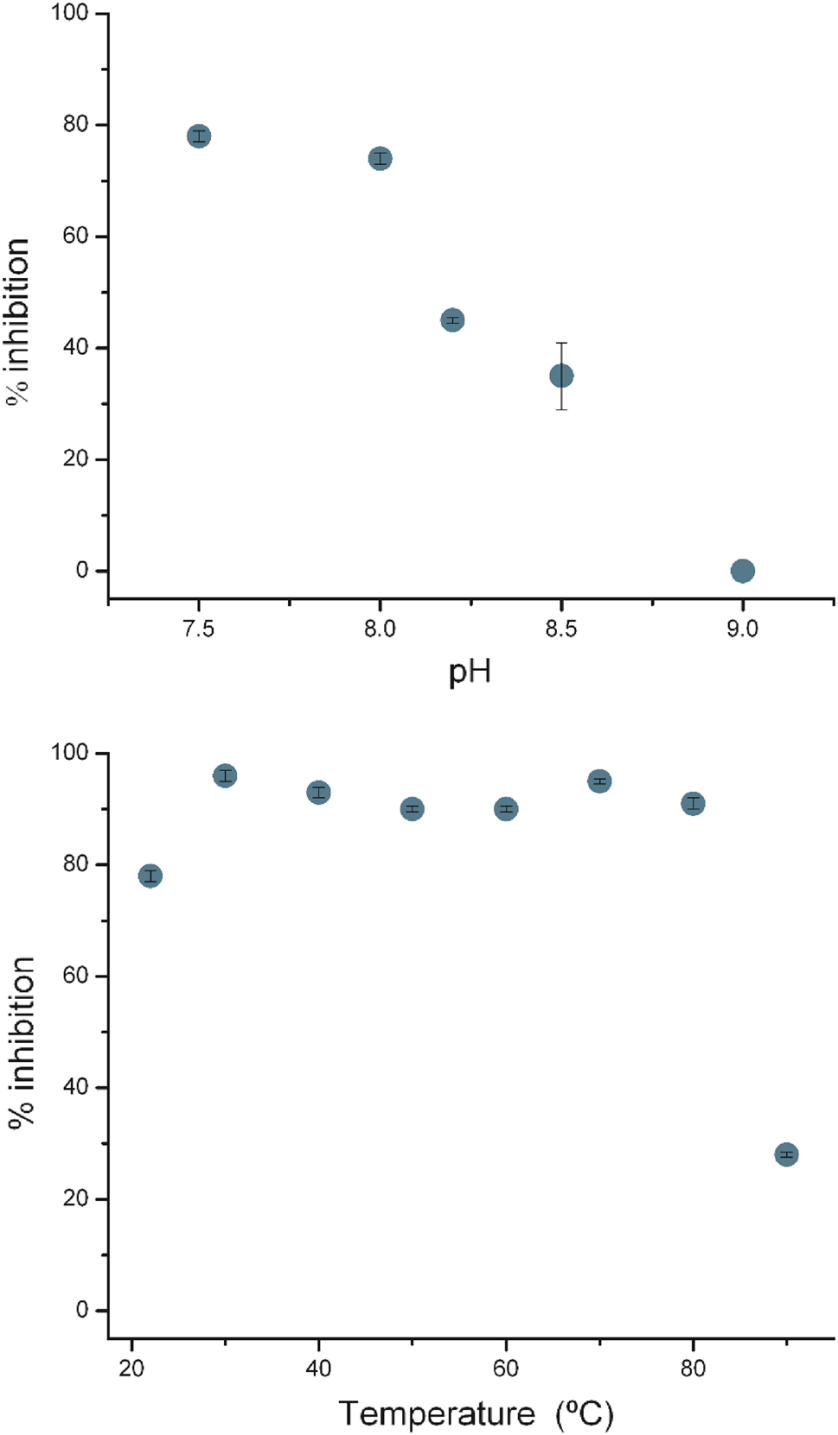
Effect of pH (top) and temperature (bottom) on *Tf*SOD activity. The experiments were performed in triplicates and the error bars represents the standard deviation of the experiments

The same amount of enzyme (concentration of 0.67 μM) used to determine the activity of *Tf*SOD (giving approximately 50% inhibition) was used to assess the pH-optimum in the pH-range 7.5–9.0 (Fig. 1). The highest degree of inhibition was found at pH 7.5, while pH 8.0 gave almost the same degree of inhibition. This is in line with a vast majority of other SODs, both in thermophilic organisms and as a general feature of this enzyme class [63, 67–69].

**Fig. 2:**
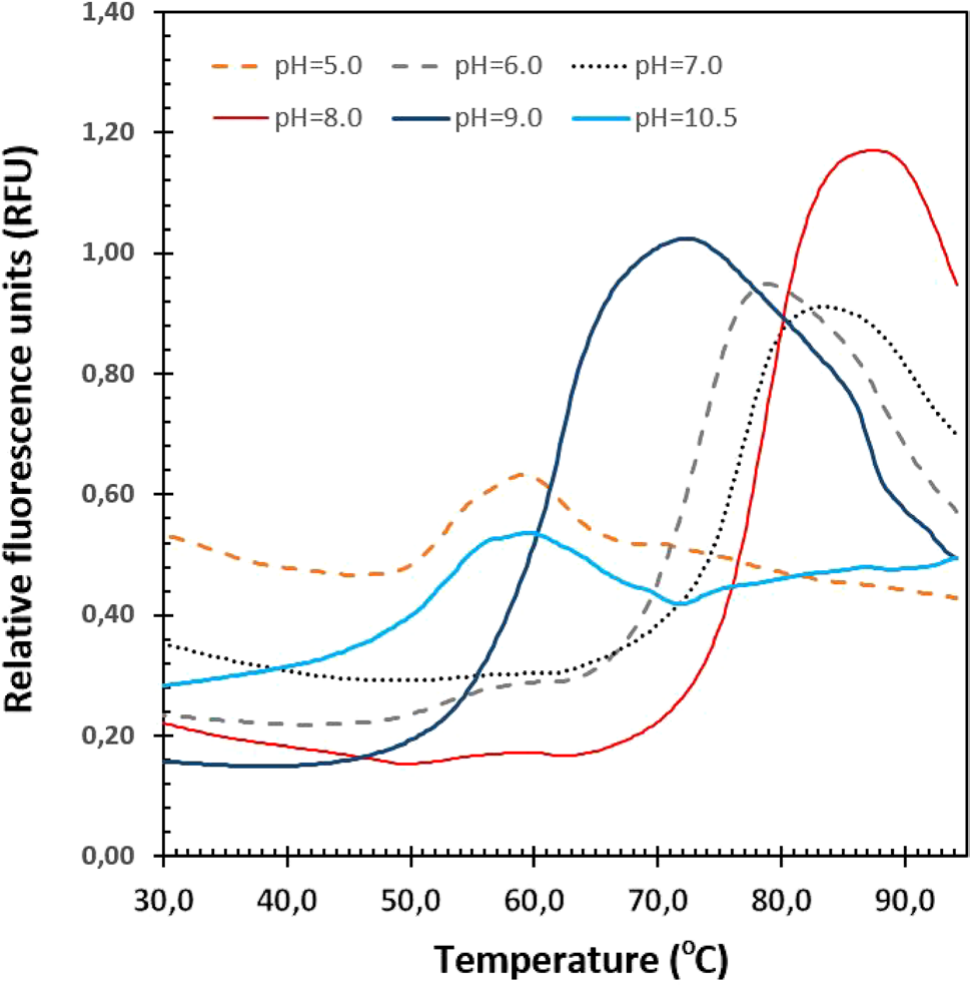
The thermostability of TfSOD is shown as fluorescence signals in different buffers at pH 5.0 (acetate; orange dashed line), pH 6.0 (MES; grey dashed line), pH 7.0 (Tris; black dotted line), pH 8.0 (Tris; red solid line), pH 9.0 (Bicine; blue solid line) and pH 10.5 (CHAPS; cyan solid line). All experiments were performed in duplicate, with error bars of 0.1–1.0 °C

Since the investigated SOD originates from the thermophilic organism *T. fusca*, the enzyme is expected to be stable at relatively high temperatures. Measuring the percent inhibition of pyrogallol autoxidation between 20 and 90 °C at the pH optimum (pH 7.5) showed that the enzyme keeps a stable, high degree of inhibition between 30 and 80 °C. The relative activity was within the interval 90–100% in this temperature range (Fig. 1). Other thermophile-derived SODs do typically exhibit optimal activity from 50 to 70 °C, like the SODs from *T. filiformis* and *Thermoascus aurantiacus* var. *levisporus* [60, 69]. Similarly to the results showed here, a SOD from the hyperthermophilic archeon *Sulfolobus solfataricus* with a relative activity ranging from 65 to 100% has a broad temperature optimum range of 20 to 100 °C [70]. If an enzyme is to be called thermostable, a high *T*_m_, or a long half-life at a temperature above the thermophile boundary for growth (> 55 °C) must be observed [40]. The *T*_m_ of *Tf*SOD was found to be 78.5 ± 0.5 °C at pH 8.0, as measured by the fluorescence–based protein thermal stability assay [47] (Fig. 2). At other pH, the melting temperatures were: 52.8 ± 1.0 °C (pH 5.0), 74.0 ± 0.8 °C (pH 6.0), 76.7 ± 0.5 °C (pH 7.0), 62.1 ± 0.1 °C (pH 9.0) and 50.8 ± 0.6 °C (pH 10.5). Thus, *Tf*SOD can be defined as a thermostable enzyme at pH 6.0–8.0.

**Fig. 3:**
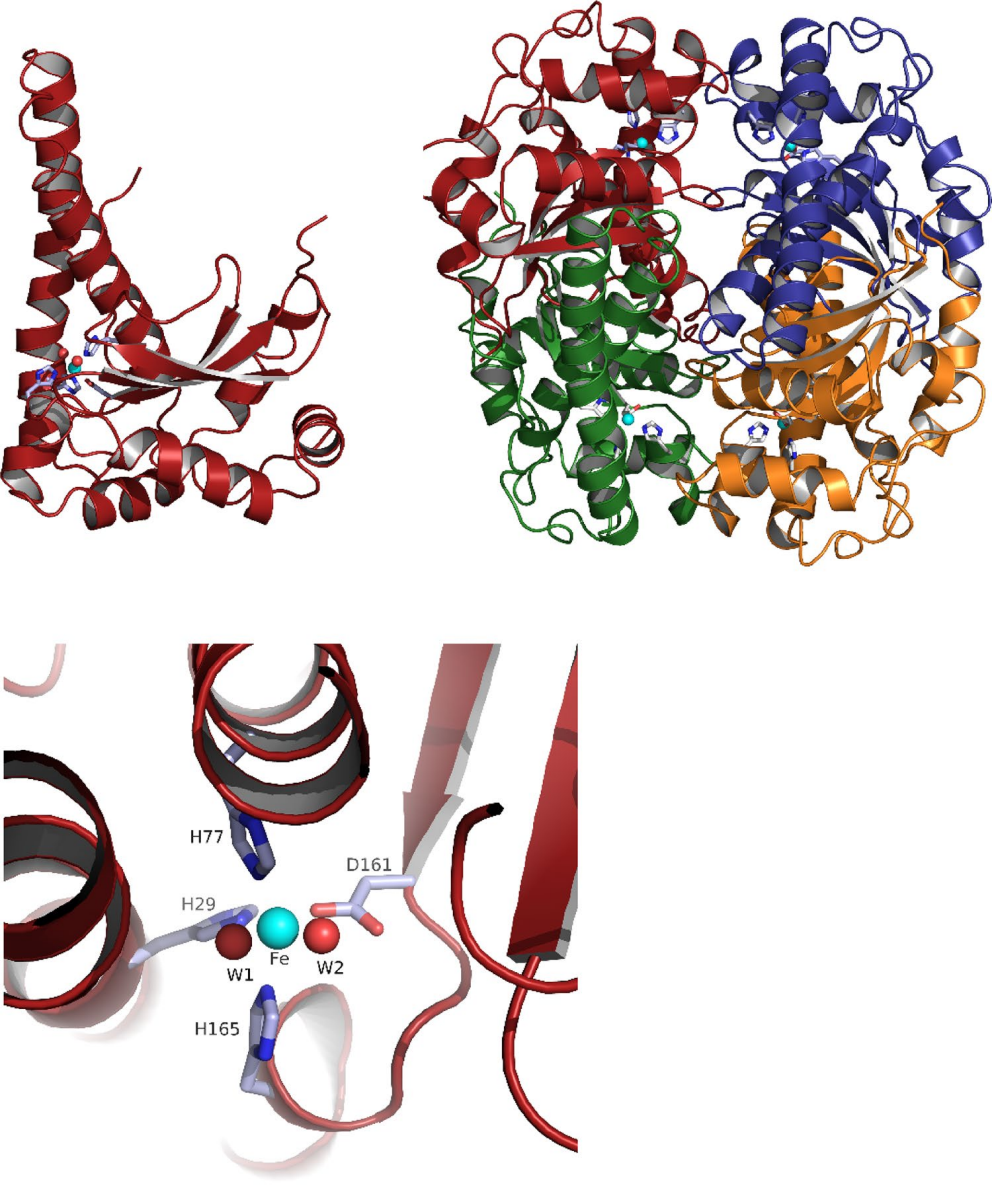
Ribbon diagram of the *Tf*SOD crystal structure as monomer (top left), tetramer with each protein chain in red, green, blue, and orange (top right), and from the metal binding site here with Fe^3+^ (cyan) and ligands (bottom)

Comparison to other SOD structures showed that *Tf*SOD has high sequence similarity towards Fe-SODs. The highly homogenous Mn- and Fe-SODs do typically occur as dimers or tetramers of ≈ 22 kDa monomer units whose two-domain fold is highly conserved [66, 71]. The C-terminal domain is in general comprised of three β-sheets surrounded by α-helices, wherein two Fe ligands are in the third β-strand and the loop that follows. The N-terminal domain is α-helical with the first and the last helices each providing one ligand to the active site Fe [59]. In this work, the crystal structure of *Tf*SOD was successfully elucidated. The crystal structure was solved by molecular replacement using the previously published crystal structure of Fe-*Pf*SOD as a starting model. The structure was determined to 1.25 Å and refined to a *R*_work_ of 11.5% (*R*_free_ = 14.1%). Statistics for diffraction data and structure refinement are summarized in Tables 1 and 2. Figure 3 shows the monomer with the three anti-parallel β-strands and D161 in the last strand. The structure has a dimer in the asymmetric unit, and the biological tetramer is generated through the crystal symmetry. The metal center in both Fe- and Mn-SOD is bound by equivalent residues, being two histidines and one aspartate as equatorial ligands, a third histidine axially coordinated, and, most probably, a hydroxide as a fifth ligand completing a trigonal bipyramidal coordination polyhedron [66]. This *Tf*SOD structure was refined with an iron bound to His29, His77, Asp161 and H165 and two solvent molecules (W1, W2). Herein W1 has higher b-factor compared to the other ligands and Fe ion, thus W1 is less accurately defined. A similar octahedral coordination was also found also found in *Propionibacterium freudenreichii* Fe^3+^ SOD (PDB ID 1BSM ref https://doi.org/10.1046/j.1432-1327.1999.00359.x), and the authors explained this from a pH value higher that 7.8 since “X-ray absorption spectroscopy measurements had shown that iron SOD could alters its coordination state from 5 to 6 at approximately pH 7.8 [66, 72].

**Table 1:** X-ray data collection statistics for the *Tf*SOD crystal structure

Diffraction source	MX Beamline BL14.1 at BESSY II
Wavelength (Å)	0.918400
Temperature (K)	100
Detector	Pilatus3 2 M
Crystal-detector distance (mm)	177.01
Exposure time per image (s)	0.3
Rotation range per image (°)	0.1
Total rotation range (°)	180
Space group	P2_1_2_1_2
*a*, *b*, *c* (Å)	98.61, 58.59, 67.94
Resolution range (Å)	25.00–1.25 (1.7–1.25)
No. of unique reflections	108,003 (9675)
Multiplicity	5.3 (1.8)
Completeness (%)	98.79 (89.74)
*R*_merge_ (%)	0.0331 (0.3475)
CC _1/2_	0.998 (0.673)
Mean 〈*I*/σ_(*I*)_〉	10.05 (1.86)
Overall *B*-factor from Wilson plot (Å^2^)	11.10

**Table 2:** Refinement statistics for the *Tf*SOD crystal structure

PDB code	
Resolution range (Å)	25.00–1.25 (1.295–1.25)
Final *R*_work_	0.1152 (0.2554)
Final *R*_free_	0.1407 (0.2930)
No. of protein chains in the asymmetric unit	2
No. of non-H atoms	
All atoms	4108
Protein	3376
Water	729
Ions	2 Fe (^3+^), 1 Cl^−^
R.m.s. deviations	
Bonds (Å)	0.007
Angles (°)	1.19
Average *B* factors (Å^2^)	
Protein	13.80
Solvent	27.70
Fe (chain A/B)	9.49/11.13
Occupancy Fe (chain A/B)	0.95/0.95
Ramachandran plot	
Most favored (%)	95.00
Allowed (%)	4.76
Disallowed (%)	0.24

Statistics for the highest-resolution shell are shown in parentheses

Even if the *Tf*SOD is resolved to high resolution at 1.25 Å it is not possible to assign one of the solvent ligands (W1, W2) as hydroxide ion from the observed electron density, still, it is an option a hydroxide ion is present as reported for other SODs.

**Fig. 4:**
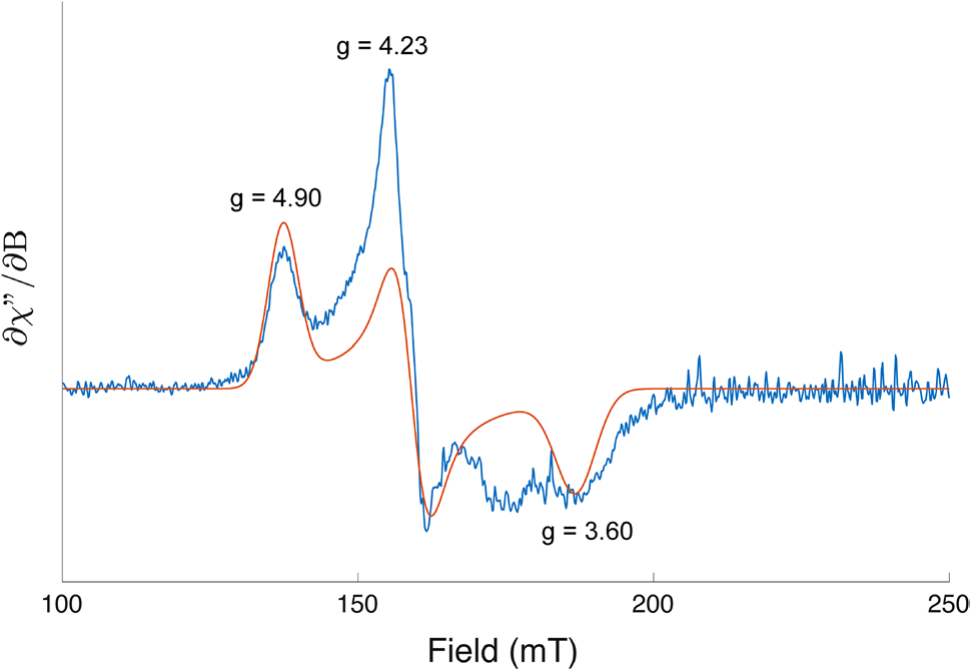
The spectrum was recorded at liquid nitrogen temperature on a sample of 200 μM *Tf*SOD in 50 mM sodium phosphate pH 7.0 and analyzed as described in the Materials and methods section. The experimental spectrum is shown in blue, the simulated spectrum is shown in red and *g* values corresponding to a ferric iron are indicated

To confirm the suggested Fe cofactor, EPR spectroscopy was conducted, giving a spectrum (Fig. 4) that was comparable to the EPR spectra of Fe-SODs from *Desulfovibrio gigas*, *Themosynechocccus elongatus*, *Escherichia coli*, and *Methanobacterium thermoautotrophicum* among other [61, 73, 74]. The EPR envelope display *g* values of 4.90, 4.23 and 3.60 that are characteristic for a high spin ferric iron in the enzyme active site. We want to point out that there is a feature in the spectrum around 175 mT that is not reproduced by the EPR calculations. The origin of the signal is unknown.

SODs must both oxidize and reduce O_2_^·^ ®. Therefore, the *E*_m_ of SODs should be half way between the *E*_m_ of these two half reactions, being approximately 360 mV [66]. Indeed, this is the case for *Tf*SOD, where the cell potential for the TfSOD-Fe^3+^/TfSOD-Fe^2+^ redox couple was determined to be 287 mV. This is in line with other Fe-SODs, e.g. Fe-SOD from *Escherichia coli*, *Pseudomonas ovalis*, and *Azotobacter vinelandii* that have *E*_m_ equaling 320 mV for the first and 230 mV for the latter two (at pH 7.0) [75, 76].

Although we observe the presence of an Fe cofactor by the crystal and EPR studies, we cannot exclude that *Tf*SOD can be cambialistic and function with an Mn cofactor as has been observed for, i.e., the SOD of *Chloroflexus aurantiacus* [64].

In conclusion, we cloned the *Tf*SOD gene from the thermophilic bacteria *T. fusca* and characterized the recombinant enzyme expressed in *E. coli* cells. The enzyme was purified by a simple two-step procedure giving a relatively good yield. Moreover, the results reveal a thermostable enzyme with a high melting temperate of 78.5 ± 0.5 °C, a broad temperature optimum range for activity (20–90 °C), and an enzyme activity optimum at pH 7.5. These feature holds a significant potential when it comes to potential future use in industrial processing. Also, the crystal structure has been determined and with the help of EPR spectroscopy it was confirmed that *Tf*SOD uses Fe as its cofactor. In high resolution *Tf*SOD crystal structure to 1.25 Å, the iron has octahedral coordinated, and the quaternary structure is tetrameric.

### Supplementary Information

Below is the link to the electronic supplementary material.

Supplementary file1 (PDF 146 KB)

## Data Availability

Raw data can be provided upon request.
