# Supplementary material for: Initial characterization of an iron superoxide dismutase from Thermobifida fusca
Source: J Biol Inorg Chem. 2023 Sep 19;28(7):689–98. doi: 10.1007/s00775-023-02019-9 (PMC10520107; doi:10.1007/s00775-023-02019-9)
Supplement: Supplementary file 1 — Supplementary file1 (PDF 146 KB) [file 775_2023_2019_MOESM1_ESM.pdf]

Initial Characterization of an Iron Superoxide Dismutase from *Thermobifida fusca*

Anne Grethe Hamre[1], Rim Al-Sadawi[1], Kirsti Merete Johannesen[2], Bastien Bisarro[1], Åsmund Røhr Kjendseth[1], Hanna-Kirsti S. Leiros[2], and Morten Sørlie[1*]

[1]*Department of Chemistry, Biotechnology and Food Science, Norwegian University of Life Sciences, PO 5003, N-1432 Ås, Norway.*

[2]*Department of Chemistry, Faculty of Science and Technology, UiT The Arctic University of Norway, N- 9037 Tromsø, Norway*

[a] Current address: *Department for Physics and Technology, Faculty of Science and Technology, UiT The Arctic University of Norway, N- 9037 Tromsø, Norway*

Supplementary information.

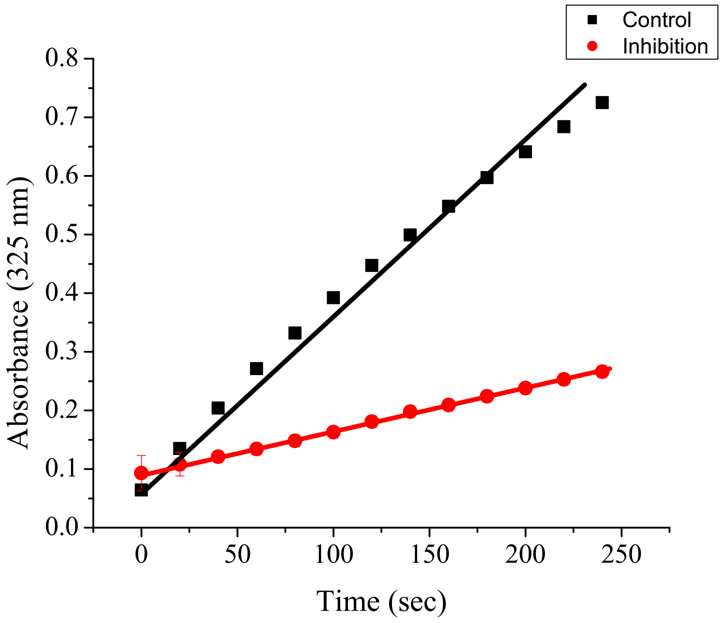

Figure S1. Time course plots of autooxidation of pyrogallol in the absence (black) and presence of *Tf*SOD in 50 mM Tris-HCl pH 8.2 containing 1 mM EDTA as measured by UV/visible spectroscopy at a wavelength of 325 nm.
